# Assessing the delivery of alcohol screening and brief intervention in sexual health clinics in the north east of England

**DOI:** 10.1186/s12889-017-4878-3

**Published:** 2017-11-17

**Authors:** C. Sullivan, N. Martin, C. White, D. Newbury-Birch

**Affiliations:** 1 Public Health England, North East Centre, Newcastle upon Tyne, UK; 2 Institute of Health & Society, Newcastle University, Newcastle upon Tyne, UK; 3 Balance, North East Alcohol Office, Durham, UK; 4 County Durham and Darlington NHS Foundation Trust, County Durham, UK; 5 School of Health and Social Care, Teesside University, Constantine Building, Middlesbrough, TS1 3BA UK

**Keywords:** Sexual health clinics, Sexual behaviour, Alcohol, Alcohol screening

## Abstract

**Background:**

Risky drinking is associated with risky sexual experiences, however the relationship between alcohol and sex is complex. The aim of the study was to assess the feasibility of delivering alcohol screening and brief interventions in genitourinary medicine (GUM) clinics. The objectives were to; understand the levels of alcohol use amongst patients; report on the number of alcohol interventions delivered; and to analyse the relationship between alcohol use with demographic data as well as diagnosed sexually transmitted infections (STIs) to see if there were any associations.

**Methods:**

All new patients attending GUM between April 2012 and March 2013 self-completed the Alcohol Use Disorder Identification Test (AUDIT) prior to their clinical consultation. Where appropriate (scoring 8+ on AUDIT) the clinician would deliver up to 2–3 min of alcohol brief intervention. Descriptive statistics, t-tests, ANOVA and logistic regression were carried out as appropriate.

**Results:**

AUDIT scores were available for 90% of all new patients (3058/3390) with an average mean score of 7.75. Of those who drank alcohol, 44% were categorised as being AUDIT positive, including 2% who had a score indicative of probable alcohol dependence (20+). 55 % (*n* = 638) of patients who screened positive on the AUDIT received a brief intervention whilst 24% (*n* = 674) of drinkers were diagnosed with a STI. Logistic regression modelling revealed that males, younger age groups and those of ‘white’ ethnicity were more likely to score positive on AUDIT. Patients classified as non-students, living in deprivation quintiles one to four and categorised as probable alcohol dependence on the AUDIT were more likely to be diagnosed with an STI.

**Conclusion:**

It is possible to embed alcohol screening into routine practice within sexual health services however further work is required to embed brief interventions particularly amongst increasing risk drinkers. If resources are limited, services may consider more targeted rather than universal alcohol screening to specific population groups. The study was undertaken in one GUM service in the North East of England and therefore findings may not be generalizable. The study did not assess efficacy of alcohol brief intervention in this setting.

## Introduction

In the United Kingdom (UK), the costs related to alcohol are €25bn (£21 bn) a year for health, welfare, employment, and criminal justice sectors as a consequence of alcohol attributable disease, injury, and violence [1]. Alcohol contributes substantially to the global burden of disease and is responsible for 2.3 million premature deaths worldwide, many of which are preventable [2]. Hazardous drinking is a repeated pattern of drinking that increases the risk of physical or psychological problems, [3] whereas harmful drinking is defined by the presence of these problems [4]. Drinking at hazardous or harmful levels are often categorised as risky drinking.

Risky drinking is also associated with risky sexual experiences. However the relationship between alcohol and sex is complex. Whilst alcohol is not directly involved in disease transmission [5] some studies from the UK [6, 7], Europe [8], and the USA [9–11] have found that it can be used to increase confidence, increase sexual arousal, enhance sexual experience, reduce inhibition, impair sexual decision-making and promote sexual behaviour. Some studies have also found that alcohol is associated with having multiple sexual partners [12], having unprotected sex, leading to higher levels of sexually transmitted infections (STIs) [5, 13, 14] and having sex that is later regretted [8]. Studies undertaken in sexual health clinics in the UK have also found that the levels of alcohol misuse are higher compared to the general population [15, 16]. However, methodological limitations exist in studies assessing the relationship between alcohol and sexual behaviour (including sexual assault) as there are no standardised measures and definitions for capturing both alcohol consumption (e.g. self-reported, frequency over time which can lead to recall bias) and sexual risk (e.g. condom use, all contraceptive use, number of sexual partners). No direct causal relationship has been proven [14, 17] however correlation has been demonstrated [13, 18, 19].

### Alcohol screening and brief intervention

Screening the adult population for risky drinking and providing feedback and brief intervention (BI) results in a reduction in the amount consumed in one in seven people [20]. The National Institute for Health and Care Excellence (NICE) recommends using the Alcohol Use Disorders Identification Tool (AUDIT) in the guidelines on alcohol-use disorders: preventing harmful drinking Public Health 24 [21, 22].

Brief interventions are not simply traditional psychotherapy delivered in a short duration of time [23, 24]. Typically they are applied to opportunistic, non-treatment seeking populations, delivered by practitioners other than addiction specialists. They largely consists of two different approaches [22]: simple structured advice which, following screening, seeks to raise awareness through the provision of personalised feedback and advice on practical steps to reduce drinking behavior and its adverse consequences; and extended brief intervention which generally involves behaviour change counselling. Extended BI introduces and evokes change by giving the patient the opportunity to explore their alcohol use as well as their motivations and strategies for change. Both forms of BI share the common aim of helping people to change drinking behavior to promote health but they vary in the precise means by which this is achieved. Typically, BIs aim to reduce alcohol consumption rather than achieve abstinence. There is a wide variation in the duration and frequency of brief alcohol interventions, however, they are typically delivered in a single session or a series of related sessions (not exceeding five sessions), lasting between two and 60 min [20]. They can be implemented by a range of practitioners in a wide variety of settings [25].

Between 20 and 30% of the population screened in primary care settings [26] and hospital settings [27] have been shown to be risky drinkers. Furthermore, it has been shown that 66% of women accessing emergency hormonal contraception in community pharmacies are risky drinkers [28].

## Background

In 2010/11 public health quality indicators were included within the contract of a local acute hospital in the North East of England as part of the Commissioning for Quality and Innovation Scheme. Given the perceived relationship between alcohol and sex, local public health strategies were keen to explore interventions to tackle multiple risk behaviours. Consequently all members of staff working in the local genitourinary medicine (GUM) clinics were trained on how to complete the AUDIT and in the delivery of BI (including brief advice and extended BI). This training was based on training previously used in primary care and accident and emergency departments in England [29, 30]. The service compromised of two clinics; one of the clinics was located within a large university city and the second clinic was located within a market town serving a large rural area.

The aim of the study was to assess the feasibility of delivering alcohol screening and BI in GUM clinics.

The objectives were:

1. to understand the levels of alcohol consumption for new patients attending the GUM clinics.
2. to report on the number of BI’s delivered as a result of a positive score following alcohol screening and whether this varied for different groups.
3. to analyse the relationship between Alcohol Use Disorders Identification Tool (AUDIT) score with age, deprivation, gender, ethnicity, sexual orientation, student identity and STI’s to see if there were any associations.

## Methods

All new patients from the two clinics were provided with an AUDIT questionnaire to self-complete prior to clinical consultation. The form was modified to include a brief statement that this was part of an overall health assessment and the ability to offer access to relevant alcohol treatment services as required. The form also included pictorial examples of units of alcoholic drinks to help self-completion. The back of the form contained space for subsequent AUDIT assessments and comments for patients returning to the clinic. Patients were brought into the clinical room by a health care worker where basic demographics were checked to confirm patient identity and a brief description of what to expect was given along with a request to self-complete the AUDIT questionnaire prior to the clinician attending.

The clinician seeing the patient would routinely check and score the AUDIT form as part of the clinical assessment. Where appropriate (indicated by scoring 8+ on the AUDIT) the clinician would deliver up to 2–3 min of BI outlining the possible consequences of excessive alcohol consumption and the relationship with sexual risks. Where appropriate a referral or information about a self-referral would be initiated to the alcohol liaison nurse based within the hospital. This was not offered to patients who identified they were already in contact with alcohol treatment services.

This current study reviewed AUDIT results for all new attendances for the period of April 2012 to March 2013. The AUDIT is considered to be the gold standard for alcohol screening in health care settings [31]. The AUDIT can be scored between 0 and 40. A score of 8+ is referred to as a ‘positive screen’ and indicates an alcohol use disorder; hazardous drinking/increasing risk drinking (score of 8–15), harmful drinking/high risk drinking (16–19) or probable dependent drinking (20+). A score of 8 or more out of a possible 40 on the AUDIT is able to detect genuine excessive drinkers (sensitivity) and to exclude false cases (specificity), and is 92% and 94%, respectively [21].

Other data collected included age, gender, sexual orientation, student status, infection details, STI tests performed, postcode of residence and positive diagnoses of STIs and infections. The STIs and infections included chlamydia; gonorrhoea; syphilis; genital herpes; genital warts; HIV; hepatitis B; hepatitis C; trichomoniasis; molluscum contagiosum; pelvic inflammatory disease (PID) and epididymitis. Using the National Statistics Postcode Lookup (ONS) patient postcodes [32] were assigned to a Lower Super Output Area (LSOA) and then in turn to a national deprivation quintile as defined by the 2010 Index of Multiple Deprivation (DCLG) [33]. As nearly half (46%) of LSOAs within the North East fall with the top 30% of most deprived LSOAs nationally, national deprivation quintiles were re-ranked within the North East region to make analyses more representative of local inequalities. A total of 51 patients were not assigned a deprivation quintile due to their recorded postcode not matching with North East postcodes included in the August 2014 postcode lookup file used at time of analysis.

### Data management

The healthcare assistants extracted data onto a standard template (Excel spreadsheet) for each patient. A reference list of patient case details was created from electronically stored records, which included case identification, demographic information, infection, testing codes and postcode of residence. Using the case ID number, hospital staff were able to reference AUDIT scores held in paper records and update the central spreadsheet.

### Data analysis

Results were analysed using SPSS v22. Descriptive statistics were used in order to characterise mean AUDIT scores for demographics such as age, gender and deprivation quintile. Statistical comparisons for mean AUDIT score were carried out using t-tests (t-values) in the case of two groups or ANOVA (F values) for more than two groups with Turkey’s Honestly Significant Difference test being used for post-hoc analyses to isolate group differences. Pearson chi-square (χ^2^) was used to test for significant differences between groups testing positive for any STI.

Three logistic regression models were created. Model 1 explored association between gender, age, student status, sexual orientation, ethnicity, deprivation and the odds ratios (OR) of being classified as AUDIT positive (8+). Model 2 looked at the same demographic variables as model 1 and introduced drinking risk group (as defined by AUDIT) to explore the ORs of being offered a BI. Model 3 explored the same demographic variables as model 2 in order to explore the ORs of being diagnosed with a STI. Only the patients who drank (scoring at least one or more on AUDIT) were included in models 1 and 3 whilst model 2 only considered patients who scored positive on AUDIT.

## Results

A total of 3390 new patients accessed the two GUM clinics from April 2012 to March 2013. Of these 47% were male (*n* = 1602) and 53% female (*n* = 1788). 9% of patients were under the age of 18 (*n* = 301), 14% were aged 18–19 (*n* = 488), 34% were 20–24 (*n* = 1136), 14% were 25–29 (*n* = 484), 9% were 30–34 (*n* = 289), 6% were 35–39 (*n* = 200) and 15% were aged 40+ (*n* = 490).

The majority (97%) of patients were of white (‘British’, ‘Irish’ or ‘other’) ethnic origin (97% (*n* = 1548) males, 97% (*n* = 1731) females) and 95% gave their sexual orientation as being ‘heterosexual’ (92% (*n* = 1472) males, 99% (*n* = 1763) females). 20% of patients defined themselves as a ‘student’ (20% (*n* = 327) males, 28% (*n* = 507) females).

Deprivation quintiles were obtainable for 98% (*n* = 3339) of patients (1 = most deprived, 5 = least deprived). 13% of patients were assigned to quintile one (*n* = 445), 20% to quintile two (*n* = 674), 23% to quintile three (*n* = 763), 16% to quintile four (*n* = 522) and 28% to quintile five (*n* = 935).

74% of patients (*n* = 2495) received the full sexual health screen for chlamydia, gonorrhoea, syphilis and HIV (81% (*n* = 1290) males, 67% (*n* = 1205) females. The three most common diagnosed STIs were genital warts: 12.5% (*n* = 423); chlamydia: 7.8% (*n* = 265); and genital herpes: 3.7% (*n* = 125).

### AUDIT scores

Full AUDIT scores were recorded for 90% of patients (*n* = 3058). 7% reported that they did not drink alcohol (7% males and 11% females). Of those patients who drank alcohol, 44% were categorised as being AUDIT positive (score of 8+) (53% (*n* = 686) males, 35% (*n* = 526) females) including 2% being categorised as having a score indicative of probable alcohol dependence (3% (*n* = 43) males, 2% (*n* = 26) females). Table 1 shows AUDIT scores broken down by age, gender, student status, sexual orientation, ethnicity and deprivation quintile. There were significant differences between mean AUDIT scores within all six of these different demographic groups with males, those aged 20–24 years, students, homosexual/bisexual patients, those who identify as white ethnic origin and people living in the least deprived quintile more likely to have a higher AUDIT score.

**Table 1:** Mean AUDIT score and numbers of drinkers broken down by drinking risk group

Demographic group	Drinkers N	AUDIT			Low risk (0–7)		AUDIT positive (8–40)		Probably dependent (20+)	
		Mean	SD	Range	N	%	N	%	N	%
Males	1297	8.79	5.10	1–40	611	47%	686	53%	43	3%
Females	1483	6.83	4.19	1–35	957	65%	526	35%	26	2%
Significant difference between gender: *t* = 11.00, *p* < 0.0005										
Under 18	236	7.15	5.46	1–35	159	67%	77	33%	10	4%
18–19	411	8.46	4.43	1–36	198	48%	213	52%	9	2%
20–24	964	8.50	4.79	1–36	452	47%	512	53%	28	3%
25–29	409	7.55	4.83	1–28	252	62%	157	38%	13	3%
30–34	237	7.20	4.64	1–30	149	63%	88	37%	7	3%
35–39	159	6.65	3.92	1–19	107	67%	52	33%	0	0%
40+	364	6.40	4.23	1–40	251	69%	113	31%	2	1%
Significant difference between age groups: F = 13.60, *p* < 0.0005										
Non-student	2064	7.61	4.89	1–40	1212	59%	852	41%	57	3%
Student	716	8.14	4.26	1–27	356	50%	360	50%	12	2%
Significant difference between student status: *t* = −2.74, *p* = 0.006										
Heterosexual	2649	7.69	4.69	1–40	1505	57%	1144	43%	63	2%
Other	131	8.82	5.47	1–35	63	48%	68	52%	6	5%
Significant difference between sexual orientation: *t* = −2.67, *p* = 0.008										
White	2706	7.80	4.74	1–40	1517	56%	1189	44%	69	3%
Black/minority/ethnic	74	5.92	4.41	1–19	51	69%	23	31%	0	0%
Significant difference between ethnicity: *t* = 3.37, *p* = 0.001										
Deprivation quintile 1	357	7.71	5.30	1–35	212	59%	145	41%	13	4%
Deprivation quintile 2	553	7.69	4.91	1–32	321	58%	232	42%	17	3%
Deprivation quintile 3	609	7.46	4.85	1–35	369	61%	240	39%	17	3%
Deprivation quintile 4	435	7.43	4.74	1–39	254	58%	181	42%	11	3%
Deprivation quintile 5	789	8.16	4.23	1–30	397	50%	392	50%	11	1%
Significant difference between deprivation quintiles (1 = most deprived): F = −2.53, *p* = 0.039										
Total	2780	7.75	4.74	1–40	1568	56%	1212	44%	69	2%

Logistic regression results showed that when controlling for other demographics, males were 2.2 times more likely (B = 0.785, *p* < 0.0005) to score positive on AUDIT when compared with females. The 18–19, 20–24 and 25–29 age groups were all significantly more likely to score positive on AUDIT when compared to the 40 plus age group with odds ratios of 2.9 (B = 1.047, *p* < 0.0005), 2.8 (B = 1.029, *p* < 0.0005) and 1.5 (B = 0.429, *p* = 0.006) respectively. Patients of ‘white’ ethnicity were also 2.1 times more likely to score positive (B = 0.738, *p* = 0.006) compared to ‘other’ ethnic groups. There were no significant ORs present between student status, sexual orientation or deprivation quintile. The full model output is shown in Table 2.

**Table 2:** Logistic regression model output for relationships between demographics and a positive score on AUDIT

	Odds ratio	P	Low 95%	Up 95%
Males	2.193	<0.0005^a^	1.864	2.58
Females^R^				
Under 18	1.354	0.11	0.934	1.964
18–19	2.85	<0.0005^a^	2.061	3.941
20–24	2.799	<0.0005^a^	2.122	3.692
25–29	1.536	0.006^a^	1.128	2.093
30–34	1.41	0.06	0.985	2.018
35–39	1.201	0.384	0.795	1.814
40 + ^R^				
Non-student^R^				
Student	1.076	0.524	0.859	1.347
Heterosexual^R^				
Other	1.118	0.555	0.772	1.621
White	2.092	0.006^a^	1.235	3.545
Black/minority/ethnic^R^				
Deprivation quintile 1^R^				
Deprivation quintile 2	1.054	0.714	0.796	1.394
Deprivation quintile 3	0.993	0.959	0.753	1.309
Deprivation quintile 4	1.041	0.792	0.774	1.4
Deprivation quintile 5	1.221	0.17	0.918	1.624

R - reference group

^a^ - significant at 99% level

### Intervention recorded as a result of the AUDIT score

Of the 1212 patients scoring positive on AUDIT, 1% (*n* = 12) were already in contact with alcohol treatment services (1%). Of the remaining 1200, it was recorded that 53% (*n* = 638) were offered a brief intervention; 49% (*n* = 502) of increasing risk drinkers; 80% (*n* = 86) of high risk drinkers and 82% (*n* = 50) of probable dependent drinkers. Of those patients offered a brief intervention 9% (*n* = 81) were further offered a referral to alcohol treatment services and declined whilst 3% (*n* = 24) accepted a referral to an alcohol treatment service. Logistic regression results revealed that the likelihood of being offered a BI was only significant for different drinking risk groups and not for any other demographic factors. Compared to increasing risk drinkers, higher risk drinkers were 4.7 times more likely to receive a BI (CI: 2.7–8.0, B = 1.538, *p* < 0.0005), whilst probably dependent drinkers were 4.6 times more likely (CI: 2.3–9.3, B = 1.527, *p* < 0.0005).

### Positive diagnoses for an STI

24% of patients (*n* = 674) who drank alcohol were diagnosed with an STI. Chi-square tests revealed that there were significant differences for drinkers being diagnosed with an STI by student status (χ2 = 18.580, *p* < 0.0005) and deprivation status (χ2 = 24.255, *p* < 0.0005). Logistic regression results showed that when controlling for demographics and drinking behaviour non-students were significantly more likely to be diagnosed with an STI with ORs of 1.6 (B = 0.44, *p* = 0.001) when compared to students. Patients classed as living in quintiles one to four were also significantly more likely to be diagnosed with an STI when compared to those living in the least deprived quintile with ORs of 1.5 for quintiles one to three and 1.4 for quintile four. There were no significant ORs present between gender, age, sexual orientation or ethnicity. However, people categorised as probably dependent on AUDIT were 1.7 times (B = 0.53, *p* = 0.041) more likely than low risk drinkers to be diagnosed with an STI. The full model output is shown in Table 3.

**Table 3:** Positive STI bivariate associations and logistic regression model output for relationships between demographics and a positive STI diagnosis (all drinkers)

	N	% testing positive for any STI	Odds ratio	P	Low 95%	Up 95%
Males^R^	1297	25%				
Females	1483	23%	0.901	0.265	0.749	1.083
No significant difference between gender and % testing positive for any STI: χ2 = 1.665, *p* = 0.106						
Under 18^R^	236	26%				
18–19	411	24%	1.077	0.701	0.738	1.571
20–24	964	26%	1.057	0.748	0.756	1.477
25–29	409	25%	0.851	0.404	0.583	1.243
30–34	237	22%	0.687	0.095	0.443	1.067
35–39	159	20%	0.632	0.073	0.383	1.043
40+	364	23%	0.756	0.166	0.51	1.123
No significant difference between age and % testing positive for any STI: χ2 = 4.613, *p* = 0.594						
Non-student	2064	26%	1.553	0.001^a^	1.193	2.021
Student^R^	716	18%				
Significant difference between student status and % testing positive for any STI: χ2 = 18.580, *p* < 0.0005						
Heterosexual	2649	24%	1.097	0.674	0.712	1.692
Other^R^	131	22%				
No significant difference between sexual orientation and % testing positive for any STI: χ2 = 0.332, *p* = 0.323						
White	2706	24%	0.808	0.458	0.46	1.419
Black/minority/ethnic^R^	74	23%				
No significant difference between sexual orientation and % testing positive for any STI: χ2 = 0.067, *p* = 0.461						
Deprivation quintile 1	357	28%	1.473	0.018^b^	1.067	2.033
Deprivation quintile 2	553	28%	1.495	0.006^a^	1.12	1.995
Deprivation quintile 3	609	27%	1.462	0.008^a^	1.103	1.937
Deprivation quintile 4	435	25%	1.389	0.033^b^	1.027	1.879
Deprivation quintile 5^R^	789	18%				
Significant difference between deprivation status and % testing positive for any STI: χ2 = 24.255, *p* < 0.0005						
Low risk^R^	1568	24%				
Increasing risk	1034	24%	1.03	0.761	0.85	1.249
Higher risk	109	23%	0.92	0.73	0.574	1.476
Probable dependence	69	38%	1.7	0.041^b^	1.021	2.83
No significant difference between risk status and % testing positive for any STI: χ2 = 7.116, *p* = 0.068						
Total	2780	24%				

R - reference group

^a^ - significant at 99% level

^b^ - significant at 95% level

## Discussion

This study set out to understand the levels of alcohol consumption of patients attending GUM clinics and adds to the literature, that patients attending sexual health clinics have higher levels of alcohol consumption than found in the general population. Furthermore, this study also identified significantly higher AUDIT scores in specific population groups accessing the sexual health service including men, those aged 20–24 years, students, homosexual/bi-sexual patients, those from a white ethnic origin and people living in the least deprived areas. Only 2% of patients in this study had a score indicative of probable alcohol dependence (3% (*n* = 43) males, 2% (*n* = 26) females) which is similar to the general population within the same geographical area [34].

The results also show that alcohol screening has been embedded into routine practice within the GUM clinics of study with 90% of patients recorded as being screened and information recorded systematically into patient records which is far greater to what has been found in other healthcare settings [35]. However, not all patients who scored positive on the AUDIT are recorded as having received a BI, in particular increasing risk drinkers (hazardous drinkers) who may have benefitted from such an intervention. The likelihood of being offered a BI was only significant for different drinking risk groups and not for any other demographic factors which suggests that sexual health practitioners were not biased by demography in their alcohol screening and delivery of BIs.

Efficacy to the intervention was not assessed in this current study, nevertheless over half of the patients who scored positive on the AUDIT (53%) were recorded as been offered a BI. Furthermore, we did find that those identified as requiring a referral onto specialist treatment for their alcohol use were identified by the clinical staff and provided with the opportunity to access treatment they may not have otherwise received. Although these numbers are low, they are higher than has been found in other settings [35] and provided the opportunity for sexual health professionals to intervene early for hazardous and harmful drinkers.

When analysing the relationship between different demographic groups with a positive diagnosis of an STI, it was non-students, those living in the four least deprived quintiles and those scoring as probably dependent on AUDIT who were significantly more likely to be diagnosed with an STI. As a result there is an argument for undertaking screening with these groups in order to attempt to reduce a patient’s level of alcohol consumption and the subsequent risk of contracting another STI in the future.

Crawford, et al. (2015) examined the clinical and cost-effectiveness of alcohol screening and BI in three sexual health clinics in London, UK. The two arm trial randomized 802 patients to either brief intervention or an advice leaflet on health and lifestyle. The adjusted mean difference in alcohol consumption at 6 months was not significantly different at −2.33 units per week (95% CI −4.69 to 0.03, *p* = 0.053) among those in the brief intervention group compared to the control arm (treatment as usual) of the trial. Unprotected sex was significantly reduced in both groups with 154 (53%) of those who received brief advice, and 178 (59%) in the control group (adjusted OR = 0.89, 95% CI 0.63 to 1.25, *p* = 0.496) [36]. However alcohol use in both groups did reduce between baseline and follow-up as did levels of unprotected sex. Similar findings in alcohol use have been found in other studies where leaflets are given as part of the control condition [29].

Therefore the question remains should alcohol screening and BI occur in this setting? To answer this question further research is needed however we believe this present study shows that it is possible to embed alcohol screening as part of routine practice by sexual health staff. As substance use is a core part of clinical assessments within GUM it is appropriate to use an existing evidence based tool. Additionally, as demonstrated in this study, with the patient self-completing the AUDIT in advance of the clinical assessment it reduces the time burden for staff. The GUM clinics involved are continuing to screen using AUDIT as part of their clinical assessment, provide BI and record this, however more work is needed to ensure everyone who screens positive are both offered and provided with an intervention.

The findings also add more depth to the literature on the levels of alcohol consumption amongst patients attending sexual health services by identifying more specific population groups who may be more at risk of risky drinking; as well as probable dependent drinkers who are more likely to also be diagnosed with an STI. Therefore sexual health services may benefit from targeted as opposed to universal screening, particularly if resources are scarce.

However this study does have limitations. The study was undertaken in one GUM service in the North East of England and therefore findings may not be generalizable to other areas. The AUDIT is a validated tool [21], however efficacy of BI in this setting has not been ascertained (and was not an objective of this study). Training did take place in how to use the AUDIT however it is a self-report measure and we cannot be sure the patients were truthful in completing the questionnaire. We did not carry out any qualitative work and only used recorded data; therefore cannot confirm whether the recording of a BI actually meant it took place or whether those that were referred to alcohol treatment services actually attended.

## Conclusion

This present study shows that it is possible to embed alcohol screening into routine practice in GUM clinics as part of clinical assessment; promoting the use of an evidence based tool and establishing pathways of care between sexual health and alcohol treatment services. However, further work is required to ascertain efficacy of BI in this setting in order to advocate for its use as either a universal or targeted public health intervention.

## Abbreviations

AUDIT: Alcohol Use Disorder Identification Test
BI: Brief Intervention
CI: Confidence interval
GUM: Genitourinary medicine
NHS: National health service
NICE: National Institute for health and care excellence
or: Odds ratio
STI: Sexually transmitted infection
UK: United Kingdom
